# Differential Serum and Urine CRP, IP-10, and TRAIL Levels in Pediatric Urinary Tract Infection

**DOI:** 10.3389/fped.2021.771118

**Published:** 2021-12-13

**Authors:** Liat Ashkenazi-Hoffnung, Gilat Livni, Oded Scheuerman, Itay Berger, Eran Eden, Kfir Oved, Liran Shani, Gali Kronenfeld, Einav Simon, Olga Boico, Roy Navon, Tanya M. Gottlieb, Eran Barash, Meital Paz, Yael Yuhas, Eva Berent, Shai Ashkenazi

**Affiliations:** ^1^Department of Day Hospitalization, Schneider Children's Medical Center, Petah Tikva, Israel; ^2^Pediatric Infectious Disease Unit, Schneider Children's Medical Center, Petah Tikva, Israel; ^3^Sackler Faculty of Medicine, Tel Aviv University, Tel Aviv, Israel; ^4^Department of Pediatrics A and B, Schneider Children's Medical Center, Petah Tikva, Israel; ^5^MeMed, Tirat Carmel, Israel; ^6^Adelson School of Medicine, Ariel University, Ariel, Israel

**Keywords:** host, biomarker, pyelonephritis, neonate, fever

## Abstract

**Background:** It is estimated that clinical evaluation and urinalysis are unable to diagnose >10% of urinary tract infections (UTI) in young children. TNF-related apoptosis induced ligand (TRAIL), interferon gamma induced protein-10 (IP-10), and C-reactive protein (CRP) exhibit differential expression in the blood in response to bacterial vs. viral infection. We assessed if the urinary and serum levels of these host biomarkers discriminate UTI, nephronia, and response to antibiotic treatment.

**Methods:** Hospitalized febrile children aged <18 years with suspected UTI based on abnormal urinalysis were recruited prospectively between 2016 and 2018; also, non-febrile controls were recruited. Following urine culture results and hospitalization course, participants were divided into three groups based on AAP criteria and expert adjudication: UTI, viral infection, and indeterminate.

**Results:** Seventy-three children were enrolled, 61 with suspected UTI and 12 non-febrile controls. Of the 61 with suspected UTI, 40 were adjudicated as UTI, 10 viral infection, and 11 as indeterminate. Urinary CRP and IP-10 levels were significantly higher in the UTI group (*p* ≤ 0.05). Urinary CRP differentiated UTI from non-bacterial etiology in children under and over 3 months of age, with AUCs 0.98 (95% CI: 0.93–1.00) and 0.82 (0.68–0.95), respectively. Similarly, urinary IP-10 discriminated with AUCs of 0.80 (0.59–1.00) and 0.90 (0.80–1.00), respectively. Serum CRP and IP-10 levels were significantly higher in UTI cases with nephronia (*p* ≤ 0.03). UTI-induced changes in the levels of urinary and serum biomarkers resolved during recovery.

**Conclusions:** CRP, IP-10, and TRAIL represent biomarkers with potential to aid the clinician in diagnosis and management of UTI.

## Introduction

Urinary tract infection (UTI) is a common illness in children that may lead to renal scarring (1, 2). In infants and young children, the possibility of renal damage after infection is considered to be higher than in older children (3); however, diagnosis and determination of infection severity in this age group are challenging, as UTI may present with non-specific symptoms and signs such as fever, irritability, poor feeding, or poor weight gain (4, 5). Since the clinical manifestations are insufficient for diagnosis, a presumptive diagnosis of UTI in children is often based on the results of urinalysis. However, the diagnostic accuracy of these tests is limited in young infants, especially in neonates, with varying sensitivity and specificity according to the parameter examined (6, 7). Urine culture is considered the gold standard for diagnosis of UTI, providing pathogen identification, as well as the antibiotic susceptibility profile; but bacterial growth may be negatively influenced by previous antibiotic therapy or by contamination during sample collection, which is especially difficult in children under 2 years. Furthermore, culture results are dependent on the threshold used to identify significant growth, cannot be differentiated from asymptomatic bacteriuria and are limited by their lengthy time to positivity (5). This diagnostic challenge contributes, on the one hand, to undertreatment or delayed treatment of UTI with its potential complications, and on the other hand to overtreatment, antimicrobial adverse effects, and increasing antibiotic resistance (8, 9).

Host protein biomarkers have intrinsic advantages that support their use to assist clinicians in the diagnosis of infection etiology, for example, their levels reflect the body's response to an invading pathogen and not to colonizers. Studies demonstrate the performance of serum host protein biomarkers for UTI diagnosis (10, 11). However, their utility has not been studied in young infants under the age of 3 months. In addition, urine biomarkers have a practical advantage since obtaining non-sterile urine samples is easier, less painful than obtaining blood samples and feasible in office settings. Of the small number of studies that have investigated the role of urine markers in the diagnosis of UTI in children (12–17), urinary retinol-binding protein (RBP), Clara cell protein (CC16), N-acetyl-beta-glucosaminidase (NAG) and neutrophil gelatinase-associated lipocalin (NGAL), and kidney injury molecule-1 (KIM-1) showed potential in differentiating between UTI and other sources of fever. However studies report contradictory results (12, 18, 19) or showed relatively low diagnostic accuracy (14, 17). An important limitation of NGAL is that it is a common marker of kidney injury, thus acute or chronic disease or other conditions can increase its level along with an infectious disease (20). Indeed, none of the biomarkers examined have made the transition to the routine clinical arena (21).

C-reactive protein (CRP) has been extensively studied as a serum marker of inflammation; nevertheless, the diagnostic value of its urinary levels has been scarcely evaluated (12). CRP is one of the three serum proteins integrated into a novel serum host-response signature. Together with tumor necrosis factor-related apoptosis-inducing ligand (TRAIL) and interferon gamma-induced protein-10 (IP-10), this host signature was validated for differentiating between bacterial and viral source of fever (called the BV signature) (10, 11). However, the diagnostic performance of these biomarkers in the urine for UTI has not been previously studied. Another data gap that has not been previously addressed relates to the kinetics of these biomarkers in children treated for UTI.

Here we conducted a prospective clinical study to evaluate the host protein biomarkers CRP, IP-10, and TRAIL in serum and in urine for their potential to aid in the diagnosis of UTI in children, including young infants under 90 days old. The dynamics of the biomarker levels during UTI treatment were also investigated.

## Methods

### Study Design and Population

Participants were recruited prospectively from April 2016 to January 2018 at the Schneider Children's Medical Center, a tertiary-care 270-bed pediatric hospital located in Israel. Eligible infectious participants were hospitalized febrile children with a presumptive diagnosis of UTI based on an abnormal urinalysis. Inclusion criteria were age under 18 years, documented temperature ≥38°C (100.4°F), presumptive diagnosis of UTI based on abnormal urinalysis (a positive leukocyte esterase or nitrite on dipstick or ≥5 white blood cells/high power field on centrifuged urine microscopy), as recommended by the American Academy of Pediatrics (AAP) guidelines (22), and symptom duration ≤ 7 days. Exclusion criteria were therapeutic antibiotic use during the preceding 2 weeks, congenital or acquired immune-deficiency, including treatment with high-dose corticosteroids >1 mg/kg/day prednisone or equivalent in the preceding 2 weeks, monoclonal antibodies, anti-tumor necrosis factor agents, intravenous immunoglobulin, and chronic severe illnesses affecting life expectancy or quality of life. The non-infectious control group were hospitalized afebrile children, such as cases of elective admission for a surgical procedure.

Patients were categorized according to age (under 3 months vs. older age) and the appearance of complications, such as lobar nephronia, defined based on ultrasound of the urinary tract (23, 24).

The study was approved by the Institutional Review Board (approval number RMC-0273-16). Patients were enrolled in the study after written informed consent was obtained from a parent or legal guardian.

### Data and Sample Collection

For each patient, the following baseline variables were recorded: demographics, medical history, physical examination, complete blood count, chemistry panel, and urinalysis. Additional testing was performed as deemed appropriate by the treating physician, e.g., multiplex-PCR diagnostic assays for viral pathogens and radiological tests (e.g., chest X-ray or ultrasound of the urinary tract).

Study-specific blood and urine samples were collected at enrollment (days 0–2 of hospital admission), during hospital admission (days 2–3) and on discharge for measurement of the biomarkers CRP, TRAIL, and IP-10. In the case of paired sampling, the urine was collected within 6 h of the serum sample. For controls, only urine samples were collected for biomarker measurement before surgery. Disease course was recorded until hospital discharge.

### Reference Standard

The reference standard for determining bacterial vs. non-bacterial etiology was based on the adjudication of two senior pediatricians, each with more than 10 years of working experience as specialists in pediatric infectious diseases. Confirmation of UTI diagnosis was according to the AAP criteria (22). These include pyuria (positive leukocyte esterase or nitrite on dipstick or >5 white blood cells/high power field on centrifuged urine microscopy) and/or bacteriuria on urinalysis, and ≥50,000 CFUs/ml growth of an uropathogen cultured from supra-pubic aspiration (SPA), bladder catheterization or midstream urine specimen. After reviewing the microbiological results of urine cultures and hospitalization course, the experts independently classified each of the infectious participants to one of the following: (a) confirmed UTI according to the AAP criteria [bacterial infection], (b) viral infection [non-UTI], (c) indeterminate or (d) mixed viral and bacterial infection. “UTI” and “viral infection” adjudication labels required both experts to assign the same label. An “indeterminate” adjudication label was given in case of a discrepancy between the assigned diagnoses, or an assigned indeterminate diagnosis, or an assigned mixed viral and bacterial infection.

### Host-Protein Measurement and Analysis

Serum CRP was measured using either one of the following kits: Cobas-6000, Cobas-Integra-400/800, or Modular-Analytics-P800 (Roche). Urinary CRP was measured by commercial high sensitivity enzyme-linked immunosorbent assay (ELISA) (Immundiagnostik AG, Bensheim, Germany). Serum and urinary TRAIL and IP-10 were measured using a commercial ELISA kit (ImmunoXpert™; MeMed). Pending analysis, samples were stored at −70°C.

The BV signature, requiring TRAIL, IP-10, and CRP serum measurements, was calculated using ImmunoXpert™ software. Cutoffs were based on manufacturer's instructions for use, i.e., BV score <35 indicated viral infection (or other non-bacterial etiology); BV score >65 indicated bacterial infection; and 35 ≤ BV score ≤ 65 was considered equivocal. Note that the intended use for the CE marked product is patients aged 90 days and over with suspected acute bacterial or viral infection.

The urinary creatinine concentration was used to normalize biomarker measurements and account for the influence of urinary dilution. Urinary creatinine concentration (mg/dL) was measured and the ratio of urinary CRP (ng/mL), urinary IP-10 (pg/mL), and urinary TRAIL (pg/mL) to urine creatinine was calculated. The laboratory technicians conducting biomarker tests were blinded to clinical data and the adjudication label.

### Statistical Analysis

Statistical analysis was performed using python version 3.7. When comparing values between two groups, *p*-values were calculated using the non-parametric Mann-Whitney *U*-test. *p* < 0.05 was deemed statistically significant.

Receiver operator characteristic (ROC) curves were plotted to compare the performance of the urinary markers, whereby the area under the curve (AUC) served as a measure for the overall ability to discriminate UTI from non-bacterial etiology. The sensitivity and specificity at the best diagnostic cut-off were calculated for urinary CRP and IP-10.

## Results

### Patient Characterization

**Figure 1:**
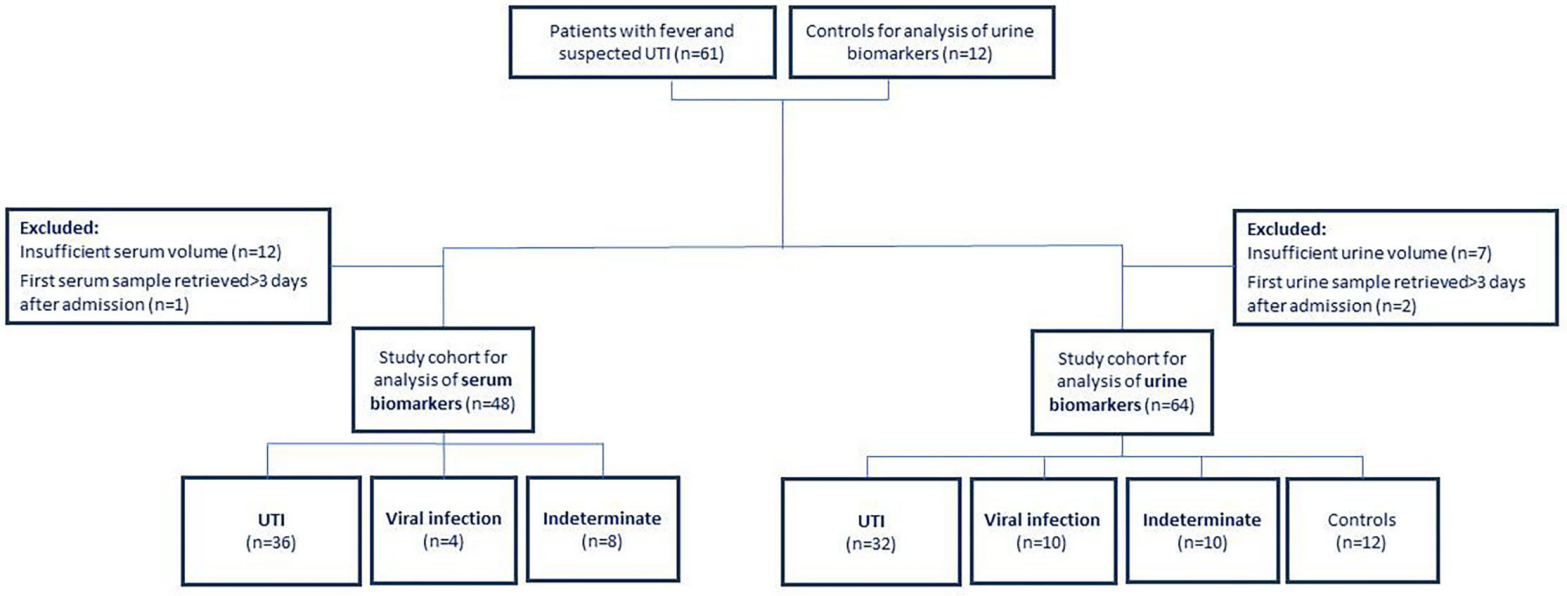
Patient recruitment flow for serum and urine biomarkers.

Sixty-one children were recruited with fever and suspected UTI and 12 healthy children, constituting a study population of 73 children aged <18 years (Figure 1). Two experts adjudicated the etiology of the 61 febrile patients: 40 were assigned as UTI infections; 10 as viral infections and the remaining 11 cases were assigned an indeterminate adjudication label. Patient characteristics are shown in Table 1.

**Table 1:** Characteristics of study population (*n* = 73).

		**UTI** **(*n* = 40)**	**Healthy** **(*n* = 12)**	**Indeterminate** **(*n* = 11)**	**Viral** **(*n* = 10)**
General information	Female	27.0 (67.5%)	5.0 (41.7%)	6.0 (54.5%)	5.0 (50.0%)
	Age (years)	0.8 (2.2)	10.5 (6.3)	2.4 (2.8)	0.2 (1.3)
Medical history	Time from symptom onset (day)	2.0 (3.2)	NA	1.0 (0.5)	4.0 (2.8)
	Maximal temperature (°C)	39.3 (1.3)	NA	39.5 (1.3)	39.0 (1.5)
	LOS (day)	5.0 (3.0)	NA	4.0 (3.0)	5.5 (4.2)
Clinical symptom	Lower respiratory	1.0 (2.5%)	0.0 (0.0%)	0.0 (0.0%)	5.0 (50.0%)
	Upper respiratory	19.0 (47.5%)	0.0 (0.0%)	3.0 (27%)	7.0 (70.0%)
	Urinary	24.0 (60%)	0.0 (0.0%)	5.0 (45.5%)	0.0 (0.0%)
	Abdominal pain	8.0 (20.0%)	0.0 (0.0%)	1.0 (9.1%)	0.0 (0.0%)
	Chills	14.0 (35.0%)	0.0 (0.0%)	3.0 (27.3%)	0.0 (0.0%)
Microbiology of urine	*Escherichia coli*	32 (80%)	NA	2 (18.2%)	0 (0%)
culture, >10^5^ bacteria/L					
	*Klebsiella* sp.	4 (10%)	NA	0 (0%)	0 (0%)
	*Proteus* sp.	1 (2.5%)	NA	0 (0%)	0 (0%)
	*Enterococcus* sp.	3 (7.5%)	NA	0 (0%)	0 (0%)
	Mixed growth	0 (0%)	NA	2 (18.9%)	0 (0%)
Urinalysis	Leukocytes, ≥250 cells/μL	35 (87.5%)	NA	6 (54.5%)	1 (10.0%)
	Leukocytes, <250 cells/μL	2 (5.0%)	NA	1 (9.1%)	7 (70.0%)
	Nitrite positive	12 (30.0%)	NA	1(9.1%)	0 (0%)
	Microscopy, ≥5 white blood cells/	37 (92.5%)	NA	8 (72.7%)	1 (10.0%)
	high power field on centrifuged urine				
	Microscopy, bacteria	27 (67.5%)	NA	4 (36.4%)	1 (10.0%)
Viral testing	Respiratory syncytial virus	0 (0%)	NA	1 (9.1%)	5 (50.0%)
	Enterovirus	0 (0%)	NA	1 (9.1%)	1 (10.0%)
	Herpes simplex virus	0 (0%)	NA	1 (9.1%)	1 (10.0%)
	Influenza virus	0 (0%)	NA	0 (0%)	1 (10.0%)
	Rota virus	0 (0%)	NA	0 (0%)	1 (10.0%)

*Categorical variables are presented as: n (%); continuous variables are presented as: median (IQR).*

*LOS, length of stay; NA, non-applicable*.

### Urinary Biomarker Levels in UTI

**Figure 2:**
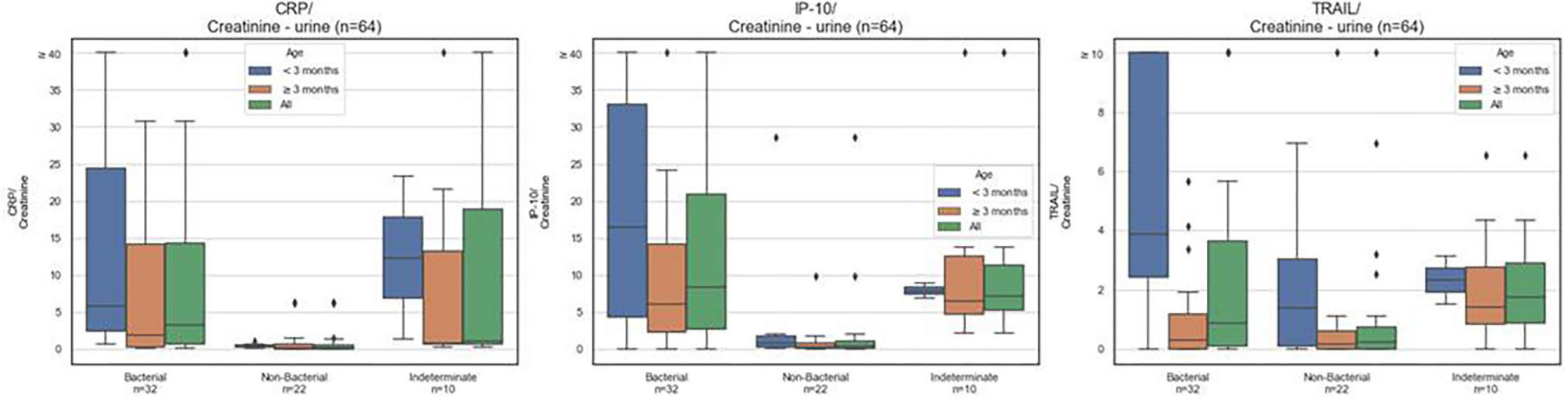
CRP, IP-10, and TRAIL levels in urine. Box plots for urine CRP (ng/mL), IP-10 (pg/mL), TRAIL (pg/mL), normalized to urine creatinine (mg/dL), measured over the entire study cohort according to assigned diagnosis: UTI, non-bacterial (viral plus healthy) or indeterminate diagnosis. The black line corresponds to group median. The box plots indicate patients with values between the 25 and 75 percentiles. Rhombus signs represent outliers.

There was no significant difference in urinary CRP, IP-10, and TRAIL levels in healthy vs. viral children (Supplementary Table 1) and so these subjects were grouped as “non-bacterial” for further analyses. The median (IQR) urinary levels of CRP (ng/mL)/creatinine (mg/dL) were significantly higher in children with UTI [*n* = 32, 3.2 (13.6)] compared to children without bacterial infection [*n* = 22, 0.2 (0.5), *p* < 0.001], also across both age groups, infants < 3 months [*n* = 11, 5.8 (22.0) vs. *n* = 6, 0.4 (0.4); *p* = 0.002], and children aged 3 months or older [*n* = 21, 1.9 (13.8) vs. *n* = 16, 0.1 (0.7); *p* = 0.001; Figure 2 and Supplementary Figure 2].

Similarly, the median (IQR) urinary levels of IP-10 (pg/mL)/creatinine (mg/dL) were significantly higher in children with UTI [*n* = 32, 8.3 (18.1)] compared to children without bacterial infection [*n* = 22, 0.3 (1.0), *p* < 0.001], also across both age groups, infants <3 months [*n* = 11, 16.4 (28.7) vs. *n* = 6, 0.9 (1.4); *p* = 0.05], and children aged 3 months or older [*n* = 21, 6.0 (11.8) vs. *n* = 16, 0.3 (0.7); *p* < 0.001]. Of note, the median urinary levels of CRP (*p* < 0.001) and IP-10 (*p* < 0.001) were significantly higher in children with UTI vs. viral infection (healthy controls excluded).

Urinary TRAIL levels were not significantly different in UTI vs. non-bacterial etiology (*p* = 0.1 for all ages; *p* = 0.1 for <3 months; and *p* = 0.5 for ≥3 months).

**Figure 3:**
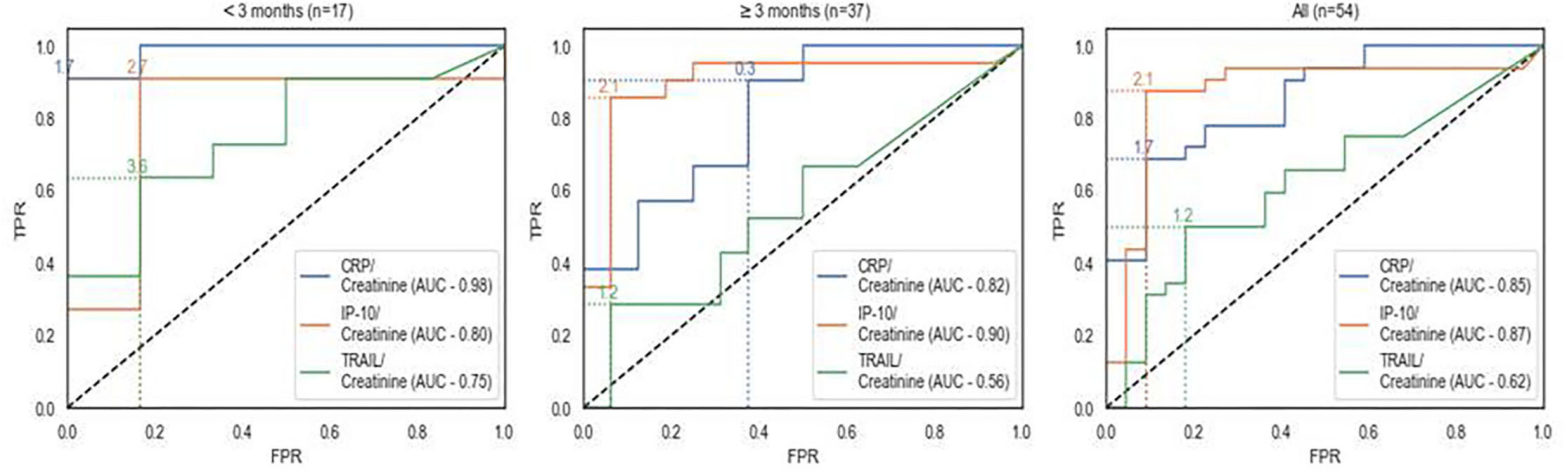
Receiver operating characteristic curve (ROC) analysis of urinary biomarkers for detecting UTI. Receiver operating characteristic curve (ROC) analysis of urinary biomarkers for detecting UTI assessed across the study population including UTI cases, viral infections and healthy children (*n* = 54). TPR, true positive rate; FPR, false positive rate; AUC, area under curve.

To examine if these urinary biomarkers differentiate between UTI and non-bacterial etiology, area under the receiver operator curve (AUC) analysis was performed, with UTI considered as positive (Figure 3). In line with the expression levels, urinary CRP displayed discriminatory potential across children of all ages with an AUC of 0.85 (95% CI, 0.75–0.95); in infants under the age of 3 months with an AUC of 0.98 (95% CI = 0.93–1.00); and in children aged 3 months or older with an AUC of 0.82 (95% CI = 0.68–0.95). Similarly, urinary IP-10 displayed discriminatory potential, with an AUC of 0.87 (95% CI, 0.78–0.96) across children of all ages; 0.80 (95% CI = 0.59–1.00) for infants aged under 3 months; and 0.90 (95% CI, 0.80–1.00) for children aged 3 months or older.

Urinary CRP (ng/mL)/creatinine (mg/dL) at a cutoff 1.7 attained the optimal sensitivity and specificity of 81.8% (59.0–100.0) and 100.0 (100.0–100.0), respectively in the subgroup of children <3 months Urinary IP-10 (pg/mL)/creatinine (mg/dL) at a cutoff of 2.1 attained the optimal sensitivity and specificity of 81.0% (64.2–97.8) and 93.8% (81.91–100.0), respectively in children ≥3 months (see Supplementary Tables 2A–C for all age groups).

The BV score was examined in the intended use population (children aged 3 months and over) for those patients with measurements available (UTI *n* = 24 and viral *n* =2); the median score was higher in children with UTI vs. viral patients, 92.0 (76.5) vs. 38.0 (35.0).

### Serum vs. Urinary Biomarker Levels

A correlation analysis between urine and serum levels of the biomarkers showed that urinary levels of CRP, TRAIL, and IP-10 did not correlate with the respective serum levels (Pearson coefficient of 0.11, 0.06, and 0.19 for CRP, TRAIL, and IP-10, respectively; Supplementary Figure 1).

Comparison of biomarker serum levels in bacterial vs. viral etiologies revealed that CRP levels are higher in UTI vs. viral infection whereas the levels of IP-10 and TRAIL are higher in viral infections (Supplementary Table 3).

### Dynamics of Biomarkers During UTI Treatment

**Figure 4:**
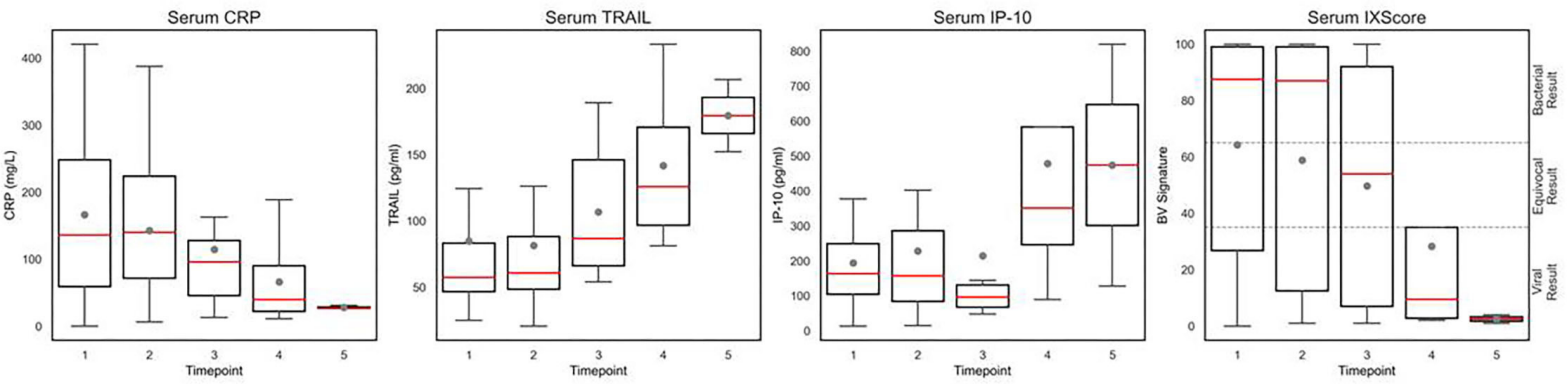
Temporal dynamics of serum CRP, IP-10, TRAIL, and the BV signature in children >90 days old with UTI. Box plots show the level of serum CRP (mg/L), IP-10 (pg/mL), TRAIL (pg/mL), and the BV signature (IX score) measured during recovery of patients >90 days old with UTI. Gray dot denotes mean level and red line denotes median level. The box plots indicate patients with values between the 25 and 75 percentiles. Timepoints 1–5 represent the sequential blood samples taken from each patient. Timepoint 1 includes samples collected at day 0 of hospital admission (*n* = 25), timepoint 2 at days 1–4 (*n* = 21), timepoint 3 at days 2–7 (*n* = 15), timepoint 4 at days 3–6 (*n* = 7), and timepoint 5 at days 4–7 (*n* = 3).

To evaluate if the biomarkers reflect response to treatment, serial samples were collected on recruitment day and at additional time points during treatment of the UTI with a course of antibiotics. The level of serum CRP decreased and serum TRAIL increased with treatment time and the BV signature decreased from a bacterial score (score >65) to a viral/other non-bacterial etiology score (score <35) (Figure 4 and Supplementary Figure 2). Similarly, the urinary levels of CRP and IP-10 decreased during antibiotic treatment of the UTI patients (Supplementary Figure 3).

### Biomarker Levels in Acute Lobar Nephronia

There were 3 UTI patients who developed acute lobar nephronia, a more serious condition typically associated with kidney damage. CRP and IP-10 demonstrated differential expression in the serum of UTI cases with and without nephronia. CRP expression (mg/L) was significantly elevated in the serum of nephronia cases aged 3 months or older (*p* = 0.03), with a median value of 382.6 (IQR 91.7) vs. 127.7 (IQR 145.6) in non-nephronia UTI patients. Similarly, median IP-10 serum levels (pg/mL) were significantly elevated in patients aged 3 months or older with nephronia (*p* = 0.03), 402.5 (IQR 37.1) vs. 164.2 (IQR 146.2). The BV signature exhibited a significantly higher score in nephronia UTI patients vs. non-nephronia patients (*p* = 0.02).

The urinary levels of the biomarkers, however, did not exhibit a significant difference in UTI with and without nephronia (*p* = 0.8 for CRP, *p* = 0.8 for IP-10, and *p* = 0.44 for TRAIL).

## Discussion

This prospective study evaluated the ability of the host protein biomarkers CRP, IP-10 and TRAIL to diagnose UTI in children under 18 years, including young infants under 3 months old. Urinary host biomarkers CRP and IP-10 were found to accurately detect pediatric UTI. Additionally, the induced levels of these biomarkers in urine and also in serum, resolved during antibiotic treatment of the UTI, raising the possibility that they could be used to monitor recovery. Lastly, serum IP-10, CRP, and BV signature scores were significantly higher in UTI patients with nephronia, suggesting these biomarkers may help to signify who has more severe disease.

The performance obtained for urinary CRP and IP-10 in UTI vs. non-bacterial etiology are superior to routine tests, such as urine leukocytes in microscopy (mean sensitivity 73% and specificity 81%) and leukocyte esterase (mean sensitivity 83% and specificity 78%) (7). Notably, these traditional tests are reported to be even less effective in children under the age of 2 years (6), which represented 56.2% of our cohort. Therefore, these host biomarkers represent potential aids to diagnose UTI in young children, an additional tool for clinicians when common clinical manifestations and laboratory parameters are insufficient. Urine biomarker measurement can be easily applied in young febrile children and this actionability may aid in reducing antibiotic underuse and overuse. The advantages of a simple, non-invasive and non-painful method of urine collection for biomarker measurements with a test attaining high specificity is especially useful in ambulatory settings to reduce unnecessary invasive workup of young children with suspected UTI.

Of note, previous studies have shown that the BV signature, which integrates the serum levels of CRP, IP-10, and TRAIL, exhibited higher diagnostic performance than its composite markers (10, 11, 25). Accordingly, the integration of urinary CRP and IP-10 levels into a urinary host signature may be an area for future research in larger groups of children.

Previously, Gorczyca et al. reported no difference in the urinary levels of IP-10 in patients with UTI compared to healthy controls (26). The contradictory findings may be ascribed to the different inclusion criteria (e.g., fever was not required) and/or to lack of normalization of IP-10 levels to urinary creatinine in the previous study, which is crucial when examining urine markers.

The lack of a significant correlation between serum and urine levels of CRP and IP-10 may be explained by the fact that they are also produced in the kidneys (27–29). This is further supported by the finding that urinary levels of CRP are higher in children with UTI than in children with extra-renal bacterial infections (12).

In line with previous reports (10, 11, 25), serum CRP, IP-10, and TRAIL were significantly differentially expressed in bacterial vs. viral etiology. In the present study, the levels of the biomarkers were also measured in infants under 3 months old and exhibited the same trend. Additionally, in the present study, for the first time, the UTI-induced biomarker levels in serum and urine and the BV signature score measured in serum were observed to exhibit a trend of resolving during antibiotic treatment. Accordingly, these host biomarker measurements have potential to serve as a guide for the clinician to personalize antibiotic therapy and shorten its duration in febrile UTI; this finding merits future study. Moreover, the biomarkers may serve to indicate the severity of infection as it was notable that the children with nephronia yielded more extreme values of the serum biomarkers and the BV signature score. Taken together, these data highlight the potential clinical value of these biomarkers not only in diagnosis but also in the management of children with UTI, since nephronia can be difficult to recognize clinically (30) but has important implications on treatment duration and follow-up.

A key strength of this study is the application of a stringent reference standard for the diagnosis of UTI composed of a unanimous expert panel diagnosis in compliance with the AAP criteria for UTI.

A limitation of the study is its relatively small sample size. Yet, we managed to include a considerable group of young infants under the age of 3 months, which was underrepresented in previous studies. Larger studies are required to validate the clinical value of urinary CRP and IP-10 as non-invasive host biomarkers for UTI and to perform a head-to-head comparison to other urinary biomarkers. Second, the study did not include children with other bacterial infections, such as bacteremia, or inflammatory states, such as Kawasaki disease. This is because the study's aim was to investigate expression of host response urinary biomarkers specifically in children with suspected UTI. The study was designed as an exploratory first step to find out if such biomarkers could be useful when evaluating a febrile child with abnormal urinalysis results, with the actionable goal being guidance of antibiotic treatment for this target population. Examination of urinary biomarkers in other infectious and non-infectious states merits future study. Third, the analysis of the temporal dynamics of urinary biomarkers during UTI treatment was hampered by missing samples during hospitalization. However, clear trends were witnessed throughout the treatment course.

## Conclusion

Incorporation of serum and urine host response biomarkers into clinical decision making may improve the ability of clinicians to diagnose and manage UTI in young children.

## Data Availability Statement

The raw data supporting the conclusions of this article will be made available by the authors, without undue reservation.

## Ethics Statement

The studies involving human participants were reviewed and approved by Institutional Review Board of the Rabin Medical Center (approval number RMC-0273-16). Written informed consent to participate in this study was provided by the participants' legal guardian/next of kin.

## Author Contributions

SA conceptualized and designed the study, took part in patient enrollment, and reviewed and revised the manuscript. LA-H participated in study design, patient enrollment, data analysis, and drafted the initial manuscript. GL, OS, and IB participated in study design, coordinated and supervised data collection, and critically reviewed the manuscript. YY and EBe designed the data collection instruments, and carried out the laboratory biomarker measurements and analyses. EE, KO, and LS conceptualized the study and reviewed the manuscript. GK, ES, and OB participated in sample analyses. RN, TG, EBa, and MP conceptualized the study, participated in data analysis, and reviewed and revised the manuscript. All authors approved the final manuscript as submitted and agree to be accountable for all aspects of the work.

## Funding

This work was supported by a research grant from MeMed that financed the index tests. The funder did not participate in study design, patient enrollment, or data collection.

## Conflict of Interest

EE, KO, LS, GK, ES, OB, RN, TG, EBa, and MP are/were employees of MeMed. SA was the PI for the research grant received from MeMed. The remaining authors declare that the research was conducted in the absence of any commercial or financial relationships that could be construed as a potential conflict of interest.

## Publisher's Note

All claims expressed in this article are solely those of the authors and do not necessarily represent those of their affiliated organizations, or those of the publisher, the editors and the reviewers. Any product that may be evaluated in this article, or claim that may be made by its manufacturer, is not guaranteed or endorsed by the publisher.
