# Supplementary material for: Differential Serum and Urine CRP, IP-10, and TRAIL Levels in Pediatric Urinary Tract Infection
Source: Front Pediatr. 2021 Dec 13;9:771118. doi: 10.3389/fped.2021.771118 (PMC8710750; doi:10.3389/fped.2021.771118)
Supplement: Supplementary file 1 [file Data_Sheet_1.pdf]

## SUPPLEMENTARY DATA

### Differential serum and urine CRP, IP-10 and TRAIL levels in pediatric urinary tract infection

Liat Ashkenazi-Hoffnung, MD,[1,3,4] Gilat Livni, MD, [2,3,4] Oded Scheuerman, MD,[2,3,4] Itay Berger, MD [2,3,4] Eran Eden,[5] Kfir Oved,[5] Liran Shani,[5] Gali Kronenfeld, [5] Einav Simon,[5] Olga Boico,[5] Roy Navon,[5] Tanya M. Gottlieb,[5] Eran Barash,[5] Meital Paz,[5] Yael Yuhas, PhD[4], Eva Berent, BSc[4], Shai Ashkenazi, MD, MSc[6]

## CONTENTS

SUPPLEMENTARY TABLES .......... 2

Supplementary Table 1. Comparison between urinary biomarker levels in viral and healthy patients. .......... 2

Supplementary Table 2A. Performance of biomarkers in urine, all ages .......... 3

Supplementary Table 2B. Performance of biomarkers in urine, < 3 months. .......... 4

Supplementary Table 2C. Performance of biomarkers in urine, ≥ 3 months. .......... 5

Supplementary Table 3. Serum expression levels of CRP, IP-10 and TRAIL. .......... 6

SUPPLEMENTARY FIGURES .......... 7

Supplementary Figure 1. .......... 7

Supplementary Figure 2. .......... 8

Supplementary Figure 3. .......... 9

## SUPPLEMENTARY TABLES

**Supplementary Table 1. Comparison between urinary biomarker levels in viral and healthy patients.**

| | **CRP (ng/mL)/Creatinine (mg/dL)** | **IP-10 (pg/mL)/Creatinine (mg/dL)** | **TRAIL (pg/mL)/Creatinine (mg/dL)** |
|---|---|---|---|
| **Viral (n=10)** | 1.66 (SD 2.68) | 0.70 (SD 0.70) | 2.22 (SD 4.38) |
| **Control (n=12)** | 0.75 (SD 1.83) | 1.20 (SD 2.88) | 0.27 (SD 0.29) |
| **P-value** | 0.21 | 0.57 | 0.95 |

**Supplementary Table 2A. Performance of biomarkers in urine, all ages.**

| | All | | |
|---|---|---|---|
| | **CRP/Creatinine** | **IP-10/Creatinine** | **TRAIL/Creatinine** |
| **Cut off** | 1.7 | 2.1 | 1.2 |
| **ROC-AUC** | 0.85 (0.75-0.95) | 0.87 (0.78-0.96) | 0.62 (0.47-0.77) |
| **Sensitivity** | 65.6 (49.2-82.1) | 84.4 (71.8-97.0) | 46.9 (29.6-64.2) |
| **Specificity** | 90.9 (78.9-100.0) | 90.9 (78.9-100.0) | 81.8 (65.7-97.9) |
| **PPV** | 91.3 (79.8-100.0) | 93.1 (83.9-100.0) | 79.0 (60.620-97.3) |
| **NPV** | 64.5 (47.7-81.4) | 80.0 (64.3-95.7) | 51.4 (34.9-68.0) |
| **n** | 54 | 54 | 54 |

ROC-AUC, receiver operating characteristic area under curve; PPV, positive predictive value; NPV, negative predictive value.

**Supplementary Table 2B. Performance of biomarkers in urine, < 3 months.**

| | <3 months | | | |
|---|---|---|---|---|
| | **CRP /Creatinine** | **IP-10 /Creatinine** | | **TRAIL /Creatinine** |
| **Cut off** | 1.7 | 2.1 | 2.7 | 3.6 |
| **ROC-AUC** | 0.98 (0.93-1.00) | 0.80 (0.59-1.00) | 0.80 (0.59-1.00) | 0.75 (0.51-0.99) |
| **Sensitivity** | 81.8 (59.0-100.0) | 90.9 (73.9-100.0) | 81.8 (59.0-100.0) | 54. (25.1-84.0) |
| **Specificity** | 100.0 (100.0-100.0) | 83.3 (53.5-100.0) | 83.3 (53.5-100.0) | 83.3 (53.5-100.0) |
| **PPV** | 100.0 (100.0-100.0) | 90.9 (73.9-100.0) | 90.0 (71.4-100.0) | 85.7 (59.8-100.0) |
| **NPV** | 75 (44.99-100) | 83.33 (53.51-100) | 71.4 (38.0-100.0) | 50.0 (19.0-81.0) |
| **n** | 17 | 17 | 17 | 17 |

ROC-AUC, receiver operating characteristic area under curve; PPV, positive predictive value; NPV, negative predictive value.

**Supplementary Table 2C. Performance of biomarkers in urine, ≥ 3 months.**

| | ≥3 months | | | |
|---|---|---|---|---|
| | CRP/ Creatinine | | IP-10/ Creatinine | TRAIL/ Creatinine |
| **Cut off** | 1.7 | 0.3 | 2.1 | 1.2 |
| **ROC-AUC** | 0.82 (0.68-0.95) | 0.82 (0.68-0.95) | 0.90 (0.80-1.00) | 0.56 (0.37-0.75) |
| **Sensitivity** | 57.1 (36.0-78.3) | 85.7 (70.8-100.0) | 81.0 (64.2-97.8) | 23.8 (5.6-42.0) |
| **Specificity** | 87.5 (71.3-100.0) | 62.5 (38.8-86.2) | 93.8 (81.9-100.0) | 93.8 (81.9-100.0) |
| **PPV** | 85.7 (67.4-100.0) | 75.0 (57.7-92.3) | 94.4 (83.9-100.0) | 83.3 (53.51-100.0) |
| **NPV** | 60.9 (40.9-80.8) | 76.9 (54.0-99.8) | 79.0 (60.6-97.3) | 48.4 (30.8-66.0) |
| **n** | 37 | 37 | 37 | 37 |

ROC-AUC, receiver operating characteristic area under curve; PPV, positive predictive value; NPV, negative predictive value.

**Supplementary Table 3. Serum expression levels of CRP, IP-10 and TRAIL.**

| | < 3 months (n=15) | | | ≥ 3 months (n=25) | | | Study population (n=40) | | |
|---|---|---|---|---|---|---|---|---|---|
| | UTI | Viral | P-value | UTI | Viral | P-value | UTI | Viral | P-value |
| CRP, mean [mg/L] (SD) | 65.4 (55.3) | 7.3 (2.0) | 0.02 | 162.9 (131.3) | 32.9 (20.6) | 0.11 | 127.7 (118.9) | 20.1 (19.0) | 0.01 |
| IP-10, mean [pg/ml] (SD) | 109.1 (63.9) | 835.5 (227.3) | 0.02 | 261.6 (273.9) | 354.2 (350.6) | 0.36 | 206.5 (232.5) | 594.9 (368.0) | 0.03 |
| TRAIL, mean [pg/ml] (SD) | 86.3 (42.6) | 390.5 (102.0) | 0.02 | 79.8 (59.6) | 94.0 (68.5) | 0.48 | 82.1 (53.6) | 242.2 (185.3) | 0.06 |

## SUPPLEMENTARY FIGURES

### Supplementary Figure 1.

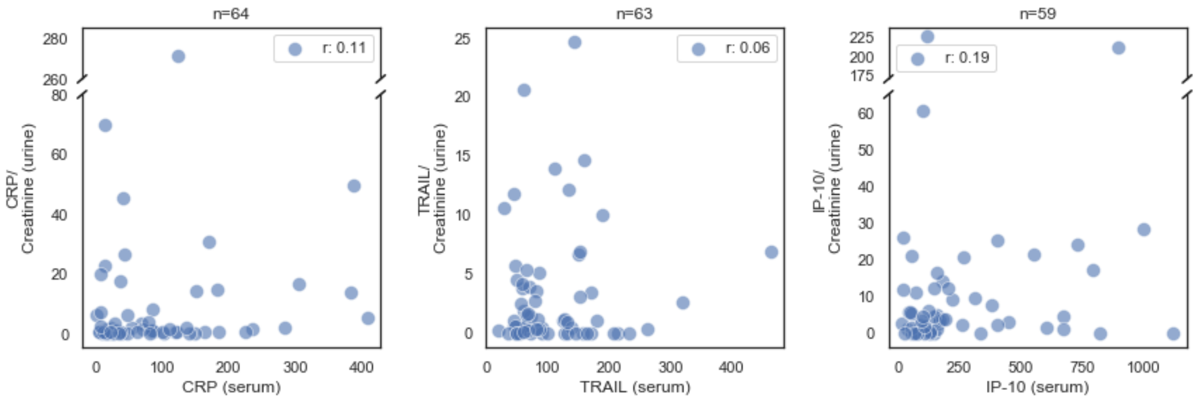


**Supplementary Figure 1. Correlation between urine and serum biomarker levels.**

**Supplementary Figure 2.**

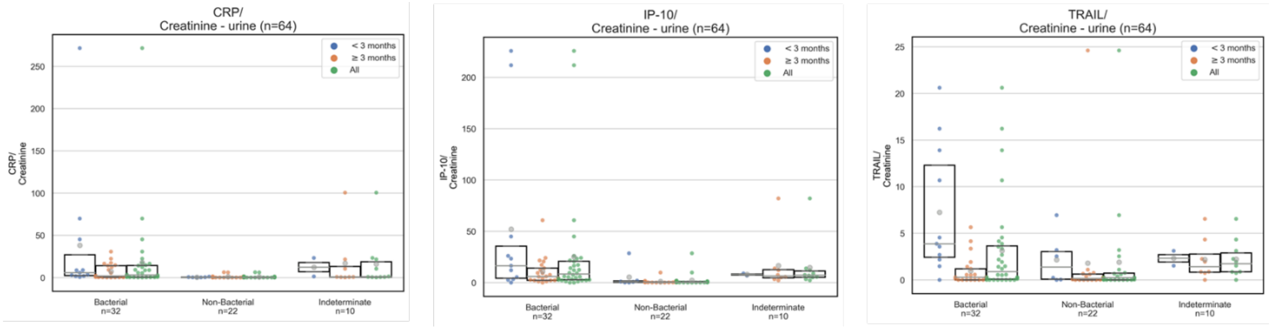


**Supplementary Figure 2. CRP, IP-10 and TRAIL levels in urine (dot plots).** The figure presents the data shown in Figure 4, here in dot plots, to show individual patient data. Dot plots for urine CRP (ng/mL), IP-10 (pg/mL), TRAIL (pg/mL), normalized to urine creatinine (mg/dL), measured over the entire study cohort according to assigned diagnosis: UTI, non-bacterial (viral plus healthy) or indeterminate diagnosis. The black line corresponds to group median. The boxes indicate patients with values between the 25 and 75 percentiles.

**Supplementary Figure 3.**

**A.**

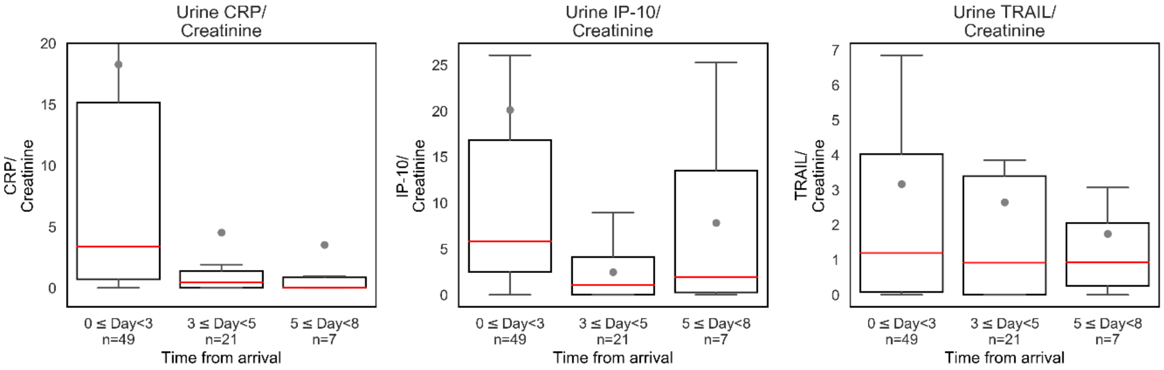


**B.**

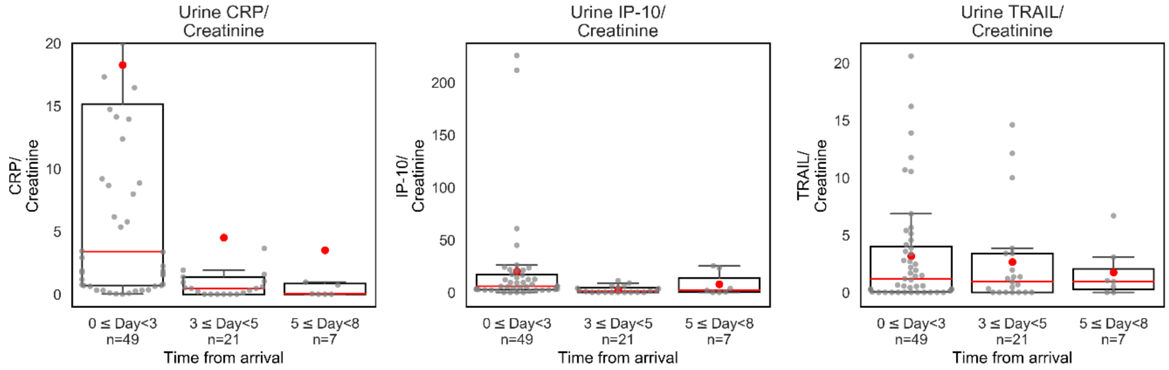


**Supplementary Figure 3. Temporal dynamics of urinary CRP and IP-10 in patients with UTI.** Box plots (A) and dot plots (B) showing the level of urinary CRP (ng/mL) and IP-10 (pg/mL), normalized to urine creatinine (mg/dL), measured during recovery of patients with UTI. In panel A, gray dot denotes mean level; in panel B, gray dots denote individual patients and red dot denotes mean level. Red line denotes median level. The box indicates patients with values between the 25 and 75 percentiles. Nine patients had two sequential samples included in the first time-frame of 0-3 days.
